# Emplacement and 3D geometry of crustal-scale saucer-shaped intrusions in the Fennoscandian Shield

**DOI:** 10.1038/s41598-019-46837-x

**Published:** 2019-07-19

**Authors:** Sebastian Buntin, Alireza Malehmir, Hemin Koyi, Karin Högdahl, Michal Malinowski, Sven Åke Larsson, Hans Thybo, Christopher Juhlin, Annakaisa Korja, Andrzej Górszczyk

**Affiliations:** 1 Department of Earth Sciences, Uppsala University, Uppsala, Sweden; 2 Institute of Geophysics, Polish Academy of Sciences, Warsaw, Poland; 3 Department of Geology, Earth Sciences Centre, Göteborg University, Göteborg, Sweden; 4 Eurasia Institute of Earth Sciences, Istanbul Technical University, Maslak, Istanbul Turkey; 5 Institute of Seismology, University of Helsinki, Helsinki, Finland

**Keywords:** Tectonics, Geology, Geophysics

## Abstract

Saucer-shaped intrusions of tens of meters to tens of kilometres across have been observed both from surface geological mapping and geophysical observations. However, there is only one location where they have been reported to extend c. 100 km laterally, and emplaced both in a sedimentary basin and the crystalline basement down to 12 km depth. The legacy BABEL offshore seismic data, acquired over the central Fennoscandian Shield in 1989, have been recovered and reprocessed with the main goal of focusing on this series of globally unique crustal-scale saucer-shaped intrusions present onshore and offshore below the Bothnian Sea. The intrusions (c. 1.25 Ga), emplaced in an extensional setting, are observed within both sedimentary rocks (<1.5 Ga) and in the crystalline basement (>1.5 Ga). They have oval shapes with diameters ranging 30–100 km. The reprocessed seismic data provide evidence of up-doming of the lower crust (representing the melt reservoir) below the intrusions that, in turn, are observed at different depths in addition to a steep seismically transparent zone interpreted to be a discordant feeder dyke system. Relative age constraints and correlation with onshore saucer-shaped intrusions of different size suggest that they are internally connected and fed by each other from deeper to shallower levels. We argue for a nested emplacement mechanism and against a controlling role by the overlying sedimentary basin as the saucer-shaped intrusions are emplaced in both the sedimentary rocks as well as in the underlying crystalline basement. The interplay between magma pressure and overburden pressure, as well as the, at the time, ambient stress regime, are responsible for their extensive extent and rather constant thicknesses (c. 100–300 m). Saucer-shaped intrusions may therefore be present elsewhere in the crystalline basement to the same extent as observed in this study some of which are a significant source of raw materials.

## Introduction

**Figure 1:**
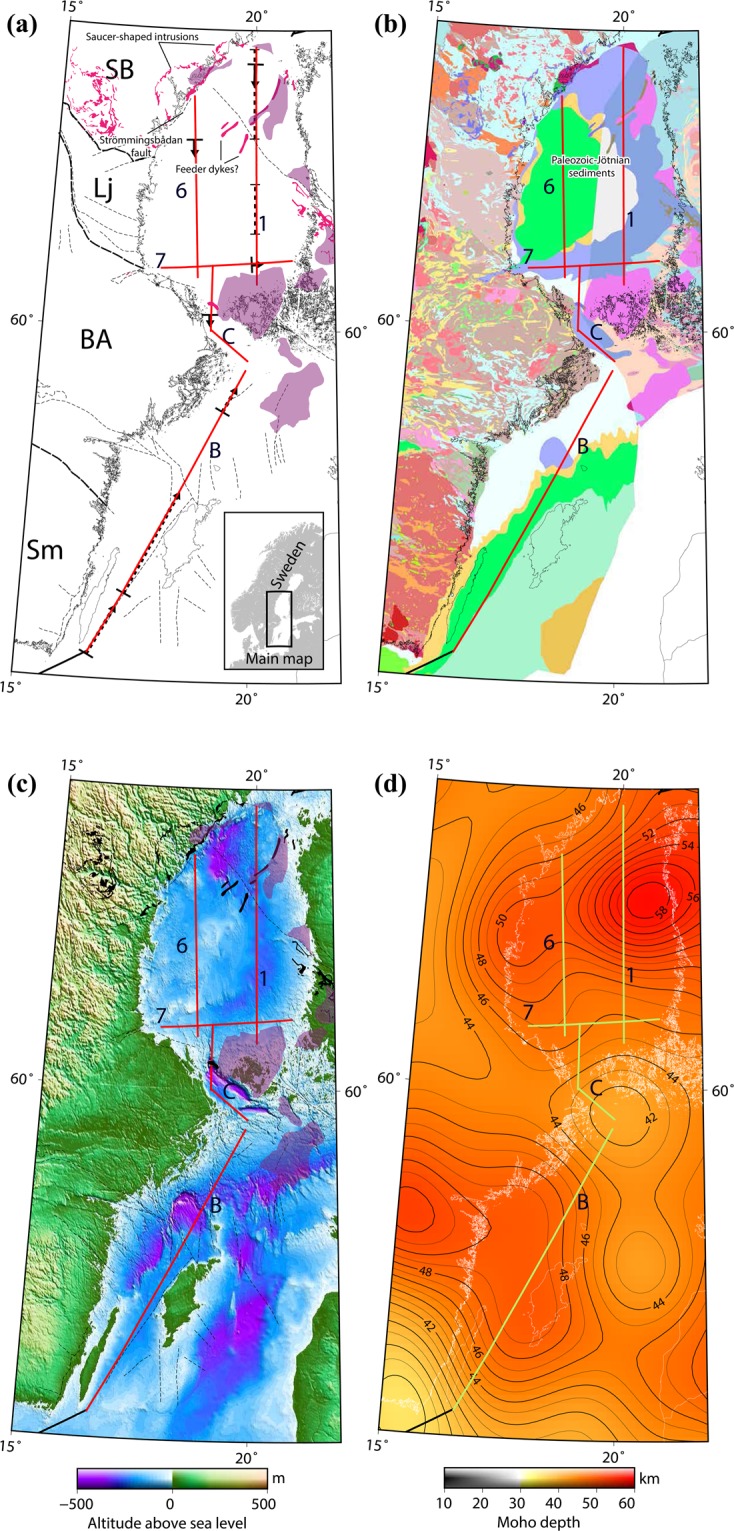
The location of the reprocessed BABEL lines with respect to (**a**) major lithotectonic units (SB, Lj, BA, and Sm), rapakivi intrusions (purple) and inferred or mapped c. 1.25 Ga dolerite intrusions of the Central Scandinavian Dolerite Group (CSDG) (pink), (**b**) general geology (based on published maps from the Geological Survey of Sweden and Finland) and location of CSDG clusters^21^, (**c**) land topography and sea bathymetry^51^, **(d**) Moho depth^52^. *Red/green lines: BABEL seismic lines; lithotectonic units: SB: Skellefte-Bothnia lithotectonic unit; Lj: Ljusdal lithotectonic unit, BA: Bergslagen lithotectonic unit, and Sm: Småland lithotectonic unit. The Generic Mapping Tools (GMT) V5.1.2 (*https://www.soest.hawaii.edu/gmt/*) was used to prepare the figures*.

Almost three decades ago (in 1988–1989), a research team from Denmark, Finland, Germany, Sweden and the United Kingdom, through the BABEL (Baltic and Bothnian Echoes from the Lithosphere) project, acquired 2,268 km of marine reflection seismic data and wide-angle refraction data in the Baltic and Bothnian Seas (Fig. 1). The main objective of the survey was to improve the understanding of the processes forming the continental crust and the juxtaposition of tectonic domains of different lithologies and metamorphic grades^1^. A major focus was therefore on imaging the Moho and its geometry and crustal thicknesses across various tectonic domains to gain information that could indicate collision or accretion of different crustal units or micro-continents during the Paleoproterozoic-Archean eras. Consequently, the original processing was geared towards imaging the lower and middle crust, leaving a major portion of the top 10 km less well imaged. This also hindered connecting the upper crust with deeper structures and past geological processes that may have affected the whole crust. The BABEL project was a significant collaborative geoscientific project in Europe seeking evidence that indicated that plate tectonic processes were active already in the Paleoproterozoic^2^. Additionally, intriguing features such as 100-km scale dolerite intrusions in the upper crust^3^ were observed and tentatively interpreted. The BABEL data have been revisited earlier by others^4–6^, however mainly for reinterpretations using the original processed data (stacked or migrated sections) when new geological and/or geophysical data became available; for example, gravitational^7^ or magnetic data^8^. The BABEL data are an extraordinary resource and have tremendous potential for improvements and for revising our understanding of past tectonic processes.

Reprocessing of legacy seismic data has shown that it is possible to improve previous results sometimes significantly by using modern software and computing power^9,10^. For the BABEL data, improvement of the image in the upper 10 km was considered likely due to the multiple strong seismic sources used and their interferences that cancelled much of the high frequencies^11^. Moreover, improving images of deeper structures should also be possible when comparing the currently used processing technology to that of the late 20^th^ century.

Here, we present reprocessing results from BABEL lines 1, 6, 7, B and C (Fig. 1), starting from raw shot gathers and processing to depth converted migrated sections. These lines were chosen due to the great interest in obtaining a better understanding of the tectonic evolution of central Fennoscandia. Lines 1 and 6 (321 and 249 km long, respectively) are parallel and strike in an approximately N-S direction at a distance of 80 km from each other in the Bothnian Sea, whereas line 7 (about 174 km long) is perpendicular to these lines and intersects them at their southern end. Lines B and C are the southward continuations of the N-S striking lines into the Baltic Sea. These lines total altogether (i.e., lines 1, 6, C and B) a 1330-km seismic section of the whole crust.

## Geology of the Central Part of the Fennoscandian Shield

The signals from the reprocessed BABEL lines (1, 6, 7, B and C) acquired in the Bothnian Sea penetrate Paleoproterozoic rocks, two major faults and subsidence-controlled depressions filled with Mesoproterozoic and younger sedimentary rocks. By convention the Mesoproterozoic sedimentary rocks in the Fennoscandian Shield are referred to as Jotnian sandstones (Fig. 1b). Preservation of these sedimentary rocks is to a large part spatially related to the 1.67–1.50 Ga rapakivi intrusions that formed in an extensional setting prior to deposition of the sediments^12^. The rapakivi magmatism is related to diapiric mantle upwelling, mafic underplating, significant crustal thinning and emplacement of the large batholiths that are present north of the Gulf of Finland on the Finnish and Russian mainland, southwestern Finland, in the Baltic and Bothnian Seas, along the coast and further inland in central Sweden^13^. Partial melting of the upper mantle is evident from mafic dykes, gabbro, and anorthosite, as well as from hybrid rocks formed by mixing of crustal and mantle derived melts. The felsic rocks originate from partial melting in the lower and middle crust, and the generated granitic magmas were emplaced as 5–10 km thick sheet-like bodies in the upper crust^14^.

Until the onset of the rapakivi magmatism, the Paleoproterozoic crust was uplifted and eroded close to the present-day levels^15^ and the Mesoproterozoic sediments were deposited unconformably on this peneplain in alluvial, fluvial or aeolian environments. In places, these units are overlain by Neoproterozoic, possibly Ediacaran, to Lower Palaeozoic sedimentary rocks (Fig. 1b). These platformal sedimentary rocks exceeds 1000 m in thicknesses while their development is attributed to a combination of syn-depositional subsidence and faulting^16,17^; the present-day distribution is related to post-depositional tectonic events. In addition to the Bothnian Sea, the Mesoproterozoic sedimentary rocks are preserved in fault-controlled basins in the Åland Sea, in the Landsort trench in the Baltic Sea and in down-faulted blocks or half-grabens on land in Sweden, Finland and western Russia, like Lake Ladoga^18^. At Lake Ladoga, sills were emplaced at 1.46 Ga, while in Sweden interlayered basaltic lava flows that reach thicknesses of up to 100 m are inferred to be related to the same magmatic event^19^. In central Sweden and SW Finland, including the Bothnian Sea dykes and sills belonging to the Central Scandinavian Dolerite Group^20^ (CSDG) occur in five separate clusters with slightly different ages, ranging between 1.27–1.25 Ga, and represent the largest manifestation of non-orogenic mafic magmatism in Europe^19–21^. They intruded both the Paleoproterozoic crust, as well as the Mesoproterozoic sedimentary rocks, providing an upper age limit for deposition of the latter. Individual intrusions range in thickness from less than ten metres to about one kilometre and in the three central clusters they are mainly present as horizontal to gently dipping saucer-shaped or oval-shaped intrusions, whereas dykes dominate the clusters to the north and south^19^. The CSDG is considered to the product of the most significant Mesoproterozoic extensional event in the Fennoscandian Shield. Isotope signatures (Hf and Nd) indicate an asthenosphere source for the magma that interacted with the subcontinental lithospheric mantle upon ascent. It has been inferred that the long-lived magmatism is related to either a mantle plume (hot spot) or discrete extensional events behind an active continental margin^21^.

Along BABEL line 1, the CSDG intrusions generate strong, concave reflections and their presence below the Bothnian Sea is further indicated by the correlation with their occurrences at the Swedish and Finnish coasts in addition to direct observations on the sea floor^22^. Until this study, the processes producing the emplacement of these intrusions, linking shallow and deeper structures observed in the seismic data, have been left unexplained. The improved seismic reprocessing results presented here are key in helping elucidate the intrusion process.

## Results

**Figure 2:**
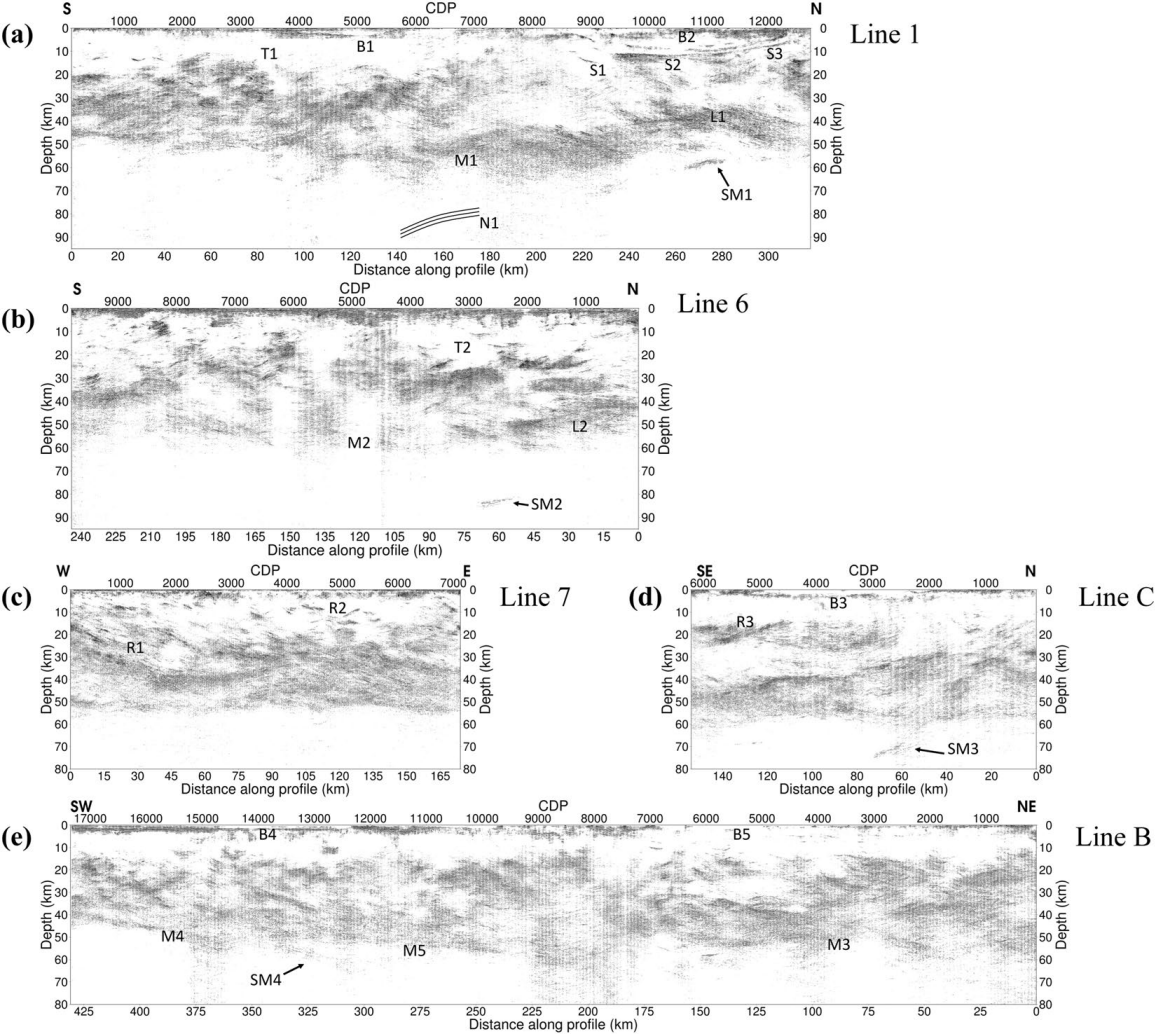
Reprocessed BABEL (**a**) line 1, (**a**) line 6, (**c**) line 7, (**d**) line B, and (**f**) line C. S1, S2, S3: saucer-shaped intrusions; B1, B2, B3, B4, B5: Basins; T1, T2: transparent regions in the crust; L1, L2: up-doming in the lower crust; M1, M2, M3, M4, M5: reflective Moho; N1: mantle reflector based on wide-angle refraction data^53^; SM1, SM2, SM3, SM4: sub-Moho reflections; R1, R2, R3: Reflections in the upper crust. The Generic Mapping Tools (GMT) V4.5.14 (https://www.soest.hawaii.edu/gmt/) was used to prepare the figures.

All the reprocessed seismic lines show improved reflectivity from near surface down to the Moho, as well as in sub-Moho reflections, (Figs 2 and Supplementary S1–S4) due to successful removal of multiples and strong source-generated noise. In the reprocessed data the Moho boundary (interpreted as the transition to a seismically transparent upper mantle) is more distinct and new shallow features in the upper crust are observed.

**Figure 3:**
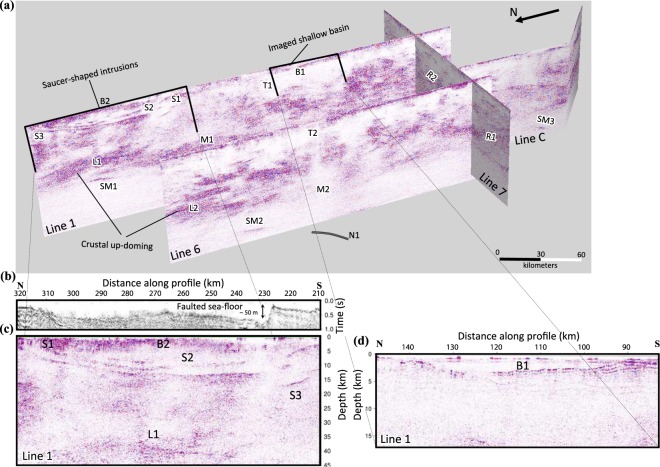
(**a**) 3D view of lines 1, 6, 7 and C, with close-ups of (**b**) the sea-floor reflection showing a rapid drop of the elevation (**c**. 50 m) associated with the region of interruption of the sill reflectivity on the southern margin of the basin and (**c**) saucer-shaped intrusions. (**d**) The Palaeozoic basin B1 revealed in the reprocessed work along line 1. S1, S2, S3: saucer-shaped intrusions; B1: Basin; T1, T2: transparent crust; L1, L2: up-doming in lower crust; M1, M2: Moho boundary; SM1-SM3: sub-Moho reflections; R1, R2: Zones of reflectivity in the upper crust.

Along BABEL line 1, and especially on its northern end, three sets of strong reflections (S1, S2, and S3) are observed from CDP 8400 to 12700 in the 1.5 km to 12 km depth range. These features are saucer-shaped (at least in the 2D seismic sections) with a sharp and steep seismically transparent zone intersecting them around CDP 9300 (Figs 2a and 3a). On the seafloor directly above this zone of transparent reflectivity there is a fault scarp (Fig. 3b). The scarp coincides with a sub-vertical dolerite intrusion, which is mapped by divers^22^ and exposed on the sea floor. A footprint of the fault (dyke filled) is also visible in the bathymetry data as well as shipborne magnetic data^22^ acquired during the same time as the seismic data were acquired. The sea floor suddenly drops by more than 50 m from south to north due to this faulting. Smaller faults can also be observed in this region based on discontinuities in the sea-floor reflection. The three sets of reflections (S1–S3) project to, or near, the surface at the margins of the Meso- to Neoproterozoic sedimentary basin and form a concentric feature (hence referred to as saucer-shaped), clearly observed in the sea-floor morphology (Fig. 1c). We argue that these reflections are primary and not multiple of one another because at their edges they appear to be similarly or even less dipping and this cannot be the case for seismic multiples. In addition, any multiple of these should occur at much later times. Reverberation is however possible but this did not show itself strong in our autocorrelation analysis of the stacked section.

On the southern half of line 1, between CDP 3600 and 5700, a basin (Palaeozoic) is observed (B1) that was previously masked in the original processing. The existence of the basin is known from the work of Winterhalter^22^ and Axberg^16^ on shallow marine seismic data. At depth, a highly reflective lower crustal up-doming is well imaged (L1) under which a clear sub-Moho reflection (SM1) dipping to the south is observed. Similarly, a sub-Moho reflection is also observed in the parallel line 6 (SM2), although deeper (c. 80 km) in the upper mantle. The lower crust is also reflective along line 6 and up-domed on its northernmost part (L2), as observed along line 1. Interestingly, along both lines 1 and 6, nearly seismically transparent areas between 5 to 12 km depths (T1 and T2) are present, with reflectivity increasing substantially below these zones.

Along line 7, two mainly opposite dipping reflectivity patterns are observed. On the westernmost part of the line, the dipping reflectivity (e.g., R1 and overlying reflections) extends to near the surface and appears to be flattening out in the lower crust at around 35–40 km depth. Where lines 1 and 6 intersect line 7 (Fig. 3a), a good correspondence between the Moho depths can be found, suggesting a reasonably well time-to-depth conversion of the data, even though different velocity functions based on the wide-angle data were used^23,24^. The eastern part of line 7 shows a higher crustal reflectivity (even distinct ones such as R2) than the western and central parts of the line.

The most striking feature along line C is an increased band of southeast-dipping reflectivity and a strong southeast-dipping sub-Moho reflection (SM3) extending down to about 80 km depth (Fig. 2d). A set of upper crustal southeast-dipping reflections is also observed on the southern part of the line (R3), where they appear to be truncated between CDP 4500–5700 (Supplementary Fig. S5).

## Interpretation and Discussion

The saucer-shaped intrusions observed along line 1 (S1, S2, S3 in Fig. 2) have been known since the initial results of the seismic data were presented^3^ and have been interpreted as intrusions in other publications too^7^. The high reflectivity of the anomalies leads us to this interpretation as well, since we have a high-impedance contrasts between the intrusion and the surrounding rocks because of their density and velocity difference. Their thickness (50–200 m) is also a further support for their strong amplitude. However, the process resulting in their emplacement both in the sedimentary rocks and in the crystalline basement over a lateral extension of 100 kilometres has been a matter of debate. A general model of emplacement was proposed by Korja *et al*.^7^. Several intrusions crosscut the present margin of the sedimentary rocks (<1.5 Ga) as evidenced by the multiple (at least four), inward dipping, oval intrusions on land that are mainly found in the crystalline basement (Fig. 1). The deepest intrusion has a diameter of c. 80 km, enclosing an area with smaller and more shallowly occurring intrusions. The reprocessed seismic sections along with the geological observations of the intrusions on land suggest therefore a ladder-shaped emplacement scenario by a network of possibly interconnected saucer-shaped intrusions of different sizes. This interconnection model is based on connectivity between the smaller saucer-shaped intrusions on land and a weak set of reflections appearing to connect two deeper intrusions (S2 and S3 reflections) in the central part of the system. These intrusions are around 200 m thick and with a nearly 45-km radius onshore. The saucer-shaped intrusions are inferred to extend to the east of the Strömmingsbådan fault scarp offshore (Fig. 1a). Roughly NE-SW intrusions are aligned along and displaced by this fault^25^. There is a bathymetric step on the sea floor, which is either the result of differential erosional or displacement during or after dyke emplacement. Faulting was likely initiated during the rapakivi magmatism, as is the case for many other faults in the Bothnian and Baltic Seas. They have been repeatedly reactivated^15^, probably until the opening of the North Atlantic.

**Figure 4:**
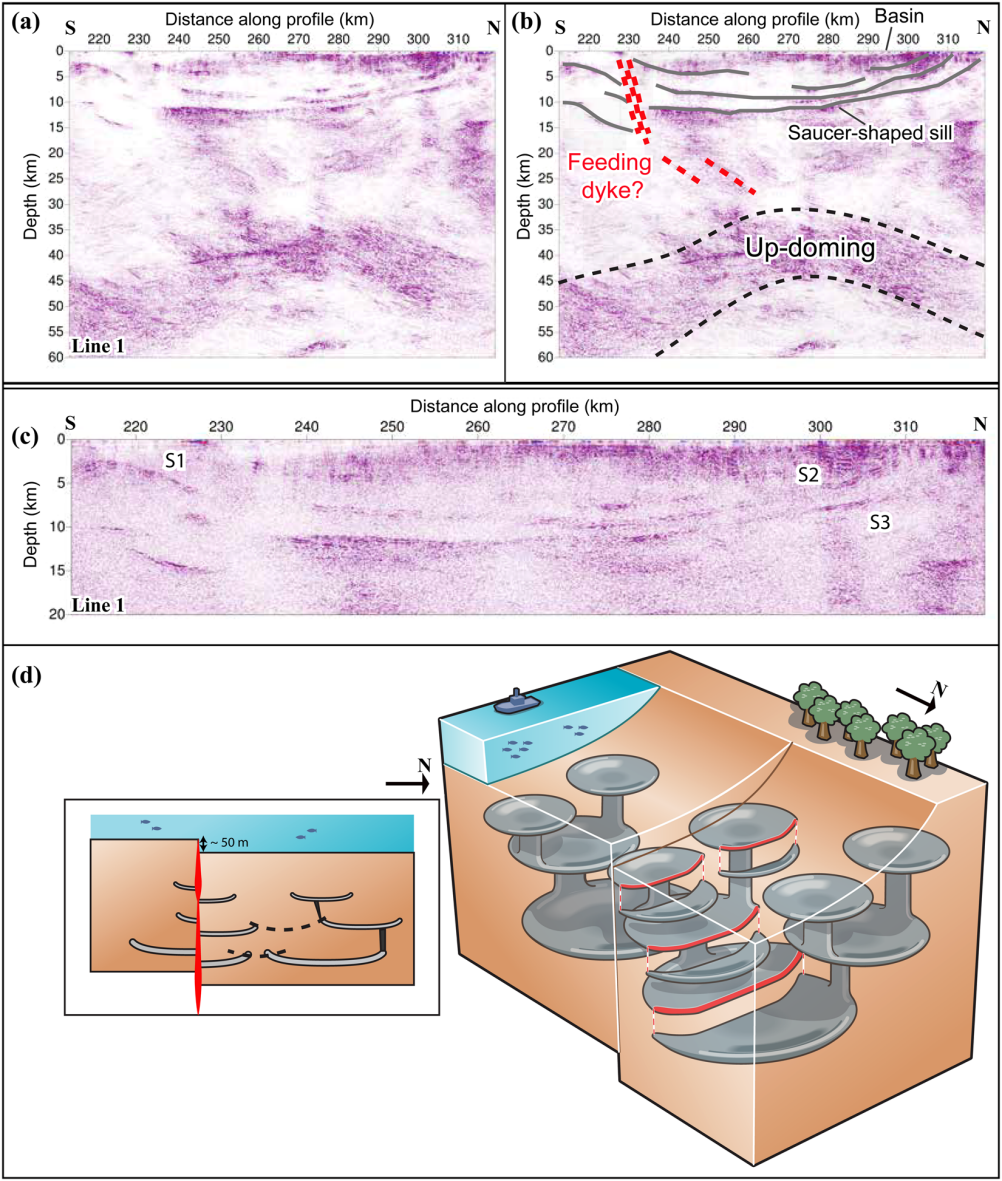
(**a**) Northern portion of line 1 where the saucer-shaped sills are imaged. (**b**) Interpretation of the sills and the lower crustal up-doming feature and one of the possible feeder dykes, (**c**) the saucer-shaped intrusions on the northern part of line 1 shown down to 20 km depth, and (**d**) schematic block diagram showing the lateral connection of the intrusions fed by dykes from a magma chamber. The red dashed line in (**b**) should be seen as just a hint that there is a connection between the feeder dyke and up-doming, but without giving an exact interpretation of their locations. Note that in 3D, only segment(s) of a feeding dyke is(are) connecting the intrusions of different levels and is(are) responsible for transport of melt to shallower levels. Sketch along the northern portion of line 1 is shown as an inset in (**d**). Depth is exaggerated twice in (**a,b**).

The saucer-shaped intrusions are interpreted to result from the same process, i.e. asthenosphere upwelling, as for the rapakivi magmatism although separated by nearly 300 My^26^. The 1.67–1.47 Ga rapakivi magmatism is related to an extensional regime and decompression melting of the upper mantle^27^. Partial melting of the middle and lower crust by heat generated from the mafic underplating produced felsic magma leaving behind a dense granulite restite^14^. Production or plumbing of the felsic melt was probably cyclic by inflation and deflation (e.g., thermal subsidence), which in the end led to several connected intrusions^28^. Emplacement of these magmas in the upper crust was associated with caldron subsidence that subsequently and successively was filled with Mesoproterozoic and younger sediments. The up-doming at the Moho (Fig. 4a,b) with a quite high velocity of 6.8 km/s to 7.1 km/s^23^ is most likely associated with the rapakivi magmatism, as it has been documented in previous studies^6,29^, but may have been enhanced by acting as a magma reservoir during the CSDG magmatism. Furthermore, the wide-angle reflection data from a previous study^23^ suggest that the lower crust is highly revebrative in front of the PmP (P-wave reflection from outer side of Moho) in the offset interval of 140–220 km. This effect has also been observed in other regions in the world^30,31^, and interpreted as a layered intrusion in the lower crust in the form of individual sills that may have formed magma chambers for the volcanic activity at the surface^32^.

While the Meso- to Neoproterozoic sedimentary rocks are only preserved locally, mainly in fault-controlled grabens, they probably covered large parts of the Fennoscandian Shield at the time of deposition^33–36^. Presently, the largest areas covered by these sedimentary rocks are in the Baltic and Bothnian Seas and Gulf of Bothnia where they are underlain to a large extent by rapakivi intrusions^37^. At least in the Baltic and Bothnian Seas these intrusions also coincide with the 1.27–1.25 Ga CSDG^19^, both emplaced in extensional settings.

In agreement with Korja *et al*.^7^, we suggest the following scenario for the formation of the dyke and sill system in the crust. The CSDG magma, with an asthenosphere source^21^, intruded through existing zones of weakness in the lithosphere, such as faults and shear zones that developed or were reactivated during extension related to the rapakivi magmatism. Through these zones enormous amount of melt migrated as dykes, and later as sills, at different levels in the upper crust. Thus, a network of several dyke zones originating from different parts of the underlying thermal anomaly is expected. The seismically transparent zone crosscutting reflections S1–S3 in the seismic section represents eventually such a network of dykes. When the pressure of the overburden and magma was nearly equal, the feeder dykes (e.g., located at CDP 9200–12600; Fig. 4a–c) began to inject melt laterally^38^, and in the case for the CSDG, into both the crystalline basement and the sedimentary rocks. In the latter, at least 300 m thick sheets of magma propagated concordantly to the bedding until the stress regime changed, leading to development of crosscutting dykes at the tip of the sills. For the larger intrusions, this occurred at the margin of the basins. Alternatively, the melt could have also used inter-basinal faults, so that they did not crosscut at the margin of the basin. These dykes were connected to and fed sills at shallower levels. In the crystalline basement, at least three major sets of reflections representing sub-horizontal intrusions are visible in the BABEL profiles (S1–S3) with their peripheral areas connected to the saucer-shaped intrusions exposed on land (Fig. 1). Dyke propagation and formation of saucer-shaped intrusions continued likely upwards through the crystalline basement to the sedimentary rocks. This upward propagation of emplacement levels was likely associated with different magmatic pulses as observed in the CSDG intrusions along the Swedish coast^25^. In this area, inflation of individual magma pulses produced gently and inward dipping dykes, which were occasionally replenished by multiple injections as shown by chilled margins. The last magma pulse is represented by crosscutting vertical dykes^25^.

We suggest that inward dipping oval-shaped intrusions along the west coast of the Bothnian Sea are related to the strong reflections observed along lines 1 and 6. These reflections show that the intrusions are located at different levels, have oval shapes of variable sizes and are connected with each other by feeder dykes (Fig. 4d). The saucer-shaped intrusions at different levels are not vertically centred, suggesting that the upward propagating tip of the deeper intrusions acted as feeder dykes to relatively younger shallower dipping dykes and sills at successively higher levels. As such, each group of saucer-shaped intrusions, including the sills, may have had separate feeder dykes that may have been tapped from the same magma chamber at the base of the lower crust that was continuously being recharged

A major fault, located offshore close to the Swedish coast, was initiated and/or reactivated during emplacement of the saucer-shaped intrusions, displacing the dykes and sills and their Mesoproterozoic sedimentary host-rocks for over 2 km^22^. Movements along this fault and continuous subsidence of the basin preserved these rocks offshore (seen along northern portions of lines 1 and 6), explaining why the saucer-shaped intrusions on land are mainly encountered in the crystalline basement. The fact that the sill reflectors display a general gentle dip towards the zone of dykes and faults (crosscutting transparent zone) may be a further indication that the fault zone and those near the shoreline must have been active during and even after the emplacement of the sills.

The saucer-shaped appearance of the intrusions is less likely to be due to post-emplacement tectonics, i.e. deflation of the underlying and feeding magma chamber, because (1) it would require deflation of many individual magma chambers at different levels, or (2) deflation of a hot-spot-scale to form such an extensive (~100-km lateral extent) saucer-shaped intrusions, which would have resulted in a massive LIP-scale melt; an interpretation, which is difficult to argue for with the current evidences available on surface geology, and (3) deflation would not suit presence of overlapping and cross-cutting sills, which have different center points and curvature.

While we could argue that the spatial relationship between the saucer-shaped intrusions and sedimentary basin(s) might support a generic relationship between the two, as has been suggested elsewhere^39,40^, several reasons led us to reject this scenario. First, the intrusions are not only confined to the sedimentary basins, on the contrary they are mainly emplaced in the crystalline basement. These inward dipping intrusions show undulated contacts as opposed to those found in the sedimentary rocks. Second, there are smaller saucer-shaped intrusions onshore that do not follow the outline of the subsided basin along seismic line 1. Third, the 1.27–1.25 Ga CSDG is also emplaced as steeply dipping dykes, both in the sedimentary rocks and in the crystalline basement. Sedimentary rocks may have covered the latter at the time of the CSDG emplacement. These points suggest a multiple batch, nested, emplacement mechanism unrelated to basin formation with the intrusions connected to one another as a multi-set of 20–100-km wide saucer-shaped bodies at different levels in the upper crust. Thus, the observed saucer-shaped intrusion along BABEL line 1 is part of a nested intrusion system, which was fed by a magma chamber in the lower crust/upper mantle as indicated by the L1/SM1 features (Fig. 2). It is worth mentioning that the sedimentary basin could have contributed to the asthenosphere upwelling isostatically, however, the melt generated from this upwelling took the route that was mechanically easier to propagate upwards through and form the nested intrusions.

Different mechanisms have been proposed for emplacement of saucer-shaped intrusions. For the Golden Valley sill in South Africa, Polteau *et al*.^28^ suggested a dynamic emplacement process characterized by inflation and deflation cycles. Whereas, Galland *et al*.^41^ used modelling results to conclude that layering controls the formation of these types of intrusions and that the ones emplaced at deeper levels have larger diameters. This model is consistent with the data presented in this study, where the deeper intrusions have far larger lateral extent than the ones at shallower levels.

The saucer-shaped intrusions are likely to have formed in sequence, from deeper to shallower levels, i.e. the shallow intrusions are relatively younger and smaller in dimension and volume. On land, the intrusions are often layered and occasionally cross-cut by steeply dipping dykes with chilled margins formed by late-stage magmas that were moving to shallower levels by transecting earlier intrusions^25,42^. Magmatic layering in the up-to 350 m thick intrusions implies that the bodies cooled slowly owing to heat generated by repeated and stacked intrusions, allowing time for development of the layered structures. It has been suggested that stacked saucer-shaped intrusions may form by magma that moves from deeper intrusions that become steeper as they propagate and act as feeder dykes for the shallower bodies^43^. This scenario is often depicted in a 2-dimensional schematic illustration (Fig. 4d). However, one of the characteristics of these saucer-shaped intrusions is their oval geometry in plan view. Any feeder system generated from a deeper oval intrusion that as a whole propagates upward will less likely have an oval geometry. Such an oval dyke will break down into smaller segments as it propagates upward due to prevailing differential pressure (Fig. 4d). A smaller more curved sheet-like feeding dyke leads to the formation of a more circular-shaped intrusion, or elliptical intrusion^44^. As it propagates, the active dyke-segment may become less-curved in the plan view (Fig. 4d). A feeder dyke segment reaches shallower levels through melt pressure, which drops as it forms and feeds saucer-shaped intrusions. The space it has created will be used again as a conduit for further melt injection. Therefore, pressure drops in the opposing segment of the dyke connected to the deeper intrusion results in the plumbing system becoming inactive. Saucer-shaped intrusions do not need to be continuous (Fig. 4d) as the magma propagation is driven by the dominating stress field, which may vary laterally^45^. The intrusions found onshore west of the Bothnian Sea intersect each other, indicating an interlinking feeding system between them. They are interpreted to be smaller and emplaced at shallower levels compared to the larger intrusions depicted on the seismic profiles.

This study has two further important implications: (1) saucer-shaped intrusions with the same lateral and depth extent (>100 km) as observed in this study can be found in the crystalline basement elsewhere. Some of these intrusions like those in South Africa are significant resources of critical raw materials such as Fe, Co, Cr and Ni. For example, the saucer-shaped intrusions of this study onshore were partly mined for their iron content in the past. (2) Legacy crustal-scale seismic data are valuable and should be kept safely with adequate documentations for reprocessing as they may provide unprecedented images for new knowledge and geological understanding that may be impossible or require high cost for obtaining through new acquisition work.

## Methods

### Seismic data acquisition

For the seismic data acquisition (year 1988–1989), the S.V. Mintrop vessel from Prakla-Seismos was used. The ship towed an array of 42 airguns with a total volume of 120.6 litters at a depth of 7.5 m. A 3-km-long hydrophone streamer with a receiver group interval of 50 m was towed behind and used for seismic data recording. The streamer consisted of 60 groups of 64 hydrophones placed at a depth of 15 m in the water. To improve spatial resolution, different shot spacings were used for different lines. Shots along lines 1, 7 and B had 75 m spacing and a recording length of 25 s while along lines 6 and C, a shot spacing of 62.5 m and a recording length of 23 s or 22 s, respectively was used. Nonetheless the sampling rate was 4 ms for all the BABEL lines^23^. Table 1 details the main acquisition parameters of the survey. The acquisition of all the BABEL lines took approximately 2.5 weeks. According to the available published reports^23^, excellent quality seismic data were acquired with minimum water-wave noise and noise from other ships passing by during the survey. Much of the reported issues were associated with the used equipment. For example, defective depth controller, power failures or autopop of the airgun^46^.

**Table 1:** Main acquisition parameters of the BABEL offshore reflection seismic survey (1898–1899) for the lines reprocessed in this study^23^.

Line/Parameters	1	6	7	C	B
No. of shots	4283	3979	2322	2419	5932
Shot interval (m)	75	62.5	75	62.5	75
Profile length (km)	~321	~249	~174	~154	~431
Receiver group interval (m)	50	50	50	50	50
Receiver cable length (m)	3000	3000	3000	3000	3000
No. of channels	60	60	60	60	60
Record length (s)	25	23	25	22	25
Sampling rate (ms)	4	4	4	4	4

### Seismic data re-processing

Data were recovered in standard SEGY (Society of Exploration Geophysicists seismic data processing format) thanks to a number of individuals who transferred them from tapes to digital disks and to standard seismic processing format. However, not much information was available in the headers in terms of geometry and acquisition set-ups. A significant amount of time was spent to gather this information in order to use them for reprocessing of the data. For example, some of the files we had in the beginning were corrupted. The first few hundred shots seemed to be fine, but at some point, the traces had no amplitude and the header information was missing. After we figured out that we were not able to recover this lost information, we could luckily find the complete files, redundant files saved, in between all the 87 BABEL files without clear structuring. In order to find the right files, we had to compare the original field record number in the header with the ones in the original observer logs. Furthermore, some of the lines were split up into several files and had to be merged together into one file before we could start with the processing work. Seismic Unix was used for this purpose. Fortunately, we also had the shot coordinates given in an ASCII file in the UKOOA P1 1984 format. Given these locations of the shots and fixed hydrophone arrays and distance from the ship marine-type geometry was set up and used with a CDP (common depth point) spacing of 25 m. A conventional poststack migration was used to carefully process the data and make sure no unwanted noise can be introduced. The reprocessing focused mainly on attenuating water-surface multiples and enhancing the Moho boundary through a careful velocity analysis and noise filtering approach. Most of the processing was done using the reflection processing software GLOBE Claritas^TM^. First, poor quality shots and receivers (misfires or gun drop-outs) were found and removed from the processing flow.

During the preparation of the data, we realized an approximately 41 ms delay on the recorded data (estimated using the nearest receiver and a water velocity of 1500 m/s). This delay was compensated using a static shift before the actual processing began. Trace balancing, compensation of spherical divergence, band-pass filtering as well as a predictive Wiener deconvolution and AGC (automatic gain control) (window 3000 ms) were used to reduce the noise and enhance the reflections. Additionally, a set of FK-filtering (frequency-wavenumber) was applied to reduce the multiples. Earlier reprocessing work^4^ also showed that FK-filtering on these types of data is in most cases sufficient to improve the shallow features. A carefully conducted velocity analysis linked to NMO (normal moveout) correction helped to image near-surface features. Bandpass filter, coherency filter (FX-deconvolution: frequency space) and FK-filter were applied post-stack to suppress artefacts and noise coming from the pre-stack processing and stacking.

Before the migration, a curvelet denoising^47^ filter preserving 15% of the highest curvelet coefficients at each scale was applied to remove any remaining noise from the data and enhance the coherency of large-scale reflections. This processing step was performed with a MATLAB toolbox developed by Górszczyk *et al*.^48^ for the purpose of legacy seismic data processing and employing Discrete Curvelet Transform implementation of Candes *et al*.^49^ Finally, a finite-difference migration algorithm, which is based on an implicit 45° migration in the time domain developed by Claerbout^50^, was used for post-stack time migration using GLOBE Claritas^TM^. It provided better results compared with other tested methods such as Kirchhoff or Phase-shift migrations. For the time-to-depth conversion, published wide-angle data were utilized ranging from 5500 m/s near the surface to 7500 m/s at the Moho level around 14 s.

Additionally, different velocities were tested in the upper crust to check the effect on the apparent up-doming of the lower crust along line 1. Figure S1 (Supplementary) shows the results for different lower crust velocities. The up-doming shape is clear in all the velocities used for this. To match the depth, intersections between the lines and Moho reflectivity was used and minor depth adjustments were manually done to fix inconsistency from one line to another. Overall, not more than 3–5 km shifts were required, and this can be used as a proxy for uncertainty with respect to the Moho depth when comparing one line against another one.

In comparison to the former processing workflow^23^, the new processing scheme differs in a few stages. Different frequency windows were used for the filtering, and no down sampling was performed during the reprocessing thanks to the improved computing power, which was not available when the original processing work was conducted (Fig. S6, Supplementary). However, the main differences are the used FK-filters, to reduce multiples, and the used stacking velocities for the imaging. It is likely that the original processing only used a constant velocity for the NMO corrections therefore missing steep features near the surface (dip-velocity dependent). An overview of all the processing steps in comparison to the former one is given in Table 2.

**Table 2:** Original^23^ and reprocessing parameters used to process some of the BABEL seismic profiles used for this study.

Original processing (1990–1991)	Reprocessing (2019)
Frequency filter	
Editing	Editing
	Balancing
Resampling	
Spherical divergence compensation	Spherical divergence compensation
	Bandpass filter (5-10-60-70 Hz)
	FK-filter
Whole trace equalization	
Receiver array simulation	
Deconvolution	Wiener deconvolution (filter length of 135 ms; gap length 30 (0–3.5 s), 35 (5–23 s)
	AGC (window of 3000 ms)
NMO corrections	NMO corrections
Muting	
CDP Stacking	CDP Stacking
	Band-pass filter (5-10-60-70 Hz)
Shot and streamer correction	
FK-filter: Pass: polygonal	
Deconvolution	Coherency filtering (FX-deconvolution)
	Balance
	Curvelet denoising for random noise attenuation
	FD migration
	Time-to-depth conversion (5500 m/s to 7500 m/s)

## Supplementary information

Supplementary information
