# Supplementary material for: Emplacement and 3D geometry of crustal-scale saucer-shaped intrusions in the Fennoscandian Shield
Source: Sci Rep. 2019 Jul 19;9:10498. doi: 10.1038/s41598-019-46837-x (PMC6642171; doi:10.1038/s41598-019-46837-x)
Supplement: Supplementary file 1 — Supplementary information [file 41598_2019_46837_MOESM1_ESM.pdf]

Emplacement and 3D geometry of crustal-scale saucer-shaped intrusions in the Fennoscandian Shield

Sebastian Buntin[1], Alireza Malehmir[1,*], Hemin Koyi[1], Karin Högdahl[1], Michal Malinowski[2], Sven Åke Larsson[3], Hans Thybo[4], Christopher Juhlin[1], Annakaisa Korja[5], and Andrzej Górszczyk[2]

1. Department of Earth Sciences, Uppsala University, Uppsala, Sweden
2. Institute of Geophysics, Polish Academy of Sciences, Warsaw, Poland
3. Department of Geology, Earth Sciences Centre, Göteborg University, Göteborg, Sweden
4. Eurasia Institute of Earth Sciences, Istanbul Technical University, Maslak, Istanbul, Turkey
5. Institute of Seismology, University of Helsinki, Helsinki, Finland

*Corresponding author:
E-mail:
alireza.malehmir@geo.uu.se
Phone: +46-18-471 2335
Postal address:
Department of Earth Sciences, Uppsala University
Villavägen 16, 752 36, Uppsala, Sweden

Figure S1. Reprocessed BABEL line 1 using different velocities:
(a) 5500 m/s at 0 s, final reprocessing result, (a) 4000 m/s at 0 s, (c) 3000 m/s at 0 s, and (d) 2000 m/s at 0 s.
*The Generic Mapping Tools (GMT) V4.5.14 (*https://www.soest.hawaii.edu/gmt/*) was used to prepare the figures.*

Figure S2. Example showing different stages of processing for a shot record along line 1:
(a) raw shot, (b) after editing, balancing and spherical divergence compensation, (c) after bandpass filtering, (d) after FK-filtering, (e) after deconvolution, and (f) after AGC.

Figure S3. Example showing different stages of processing for the unmigrated seismic section of line 1:
(a) stacked section after NMO corrections, (b) after band-pass filtering, (c) FX-deconvolution, (d) after balancing, (e) after curvelet denoising, and (f) after FD-migration.

Reprocessing work and visual comparison

We compare the new reprocessed sections with the final sections of the original processing work. The original sections include pre-stack and post-stack processing works but without time-variant bandpass filtering and time-variant scaling. To provide a fair comparison, we also applied the FD migration and time to depth conversion on the final stacked data. The migrated and depth converted sections of the original work from 1990-1991 are shown in **Figure S4**. The reprocessed sections are shown in the main article.

The sub-Moho reflections SM1, SM2, SM3 are clearer visible in the reprocessed sections. Furthermore, several structures in the lower to middle crust are better distinguishable. In the uppermost part some basins like B1 or B4 are unravelled in the reprocessed work and these are totally absent in the original processing work. They were hidden by severe multiples.

Overall, the reprocessed data make it easier to follow certain structures in the subsurface. A close-up of a portion of line C is shown in **Figure S5** highlighting a clear reflectivity truncation in the middle crust, which is much better imaged in the reprocessing work than earlier studies.

Figure S4. Migrated and depth converted original final stacked sections of BABEL data: *(a) line 1, (a) line 6, (c) line 7, (d) line B, and (f) line C.*
S1, S2, S3: saucer-shaped sills; B1, B2, B3, B4, B5: Basins; T1, T2: transparent regions in the crust; L1, L2: up-doming in lower crust; M1, M2, M3, M4, M5: reflective Moho; N1: mantle reflector reported by earlier studies; SM1, SM2, SM3, SM4: sub-Moho reflections; R1, R2, R3: Reflections in the upper crust.
*The Generic Mapping Tools (GMT) V4.5.14 (*https://www.soest.hawaii.edu/gmt/*) was used to prepare the figures.*

Figure S5. A close-up showing reflection truncation along line C *suggesting different timing for their generations.*
*The Generic Mapping Tools (GMT) V4.5.14 (*https://www.soest.hawaii.edu/gmt/*) was used to prepare the figures.*

Vote map/image for comparing original and reprocessing results

In addition to the visual comparison, we also provide an overlay of the results using the so-called vote map or image. The image processing toolbox in Matlab^{TM} was used for this purpose. The grey-scaled image of the reprocessing result uses the green channel while the original section uses the red and blue channel. Therefore, the magenta region in the vote image shows where the original processing result is brighter than the reprocessed one and conversely the green regions showing brighter image in the reprocessing work. If the image is black, grey or white, all channels are the same i.e., no significant improvement is obtained. We show this only along line 1.

The vote image, shown in **Figure S6**, contains more magenta colour in the upper part of the section although appears randomly distributed. This may imply that the original processing contained much higher noise in the upper crust than the reprocessing work conducted in this study. Additionally, a green region in the vote image at distances between 100 km to 150 km in the uppermost crust suggests that the unravelled basin was not visible in the original processing work a further support for our claim in the article.

The deeper parts of the vote image contain more greenish and less magenta colours. This leads us to conclude that the reprocessing work allowed improved imaging of the deeper structures. However, because there are many regions of black and white colours, one can argue both original and the processing works reasonably well recovered these structures. This is not surprising since deeper reflections are less sensitive to a number of processing parameters as long as they are chosen adequately reasonable.

Figure S6. A vote image for quantitative comparison of the original processing versus reprocessing work along line 1 *suggesting improvements were significant on the uppermost crust and nearly similar at the deeper parts of the section. (a) Full length of line 1 and (b) portion where the sills are discussed in the main text.*
The Matlab^{TM} was used to prepare the figures.

Known magmatic saucer-shaped intrusions

Saucer-shaped intrusions are reported worldwide but most have only been observed on the surface and only a few are traceable at depth using methods such as reflection seismic. To illustrate why the saucer-shaped sills found in the BABEL seismic lines and those onshore are globally unique, we list a number of known saucer-shaped intrusions in **Table S1**.

Table S1. Known saucer-shaped intrusions reported worldwide (after Polteau et al.[1]).

Location	Size	Reference
Karoo Basin, South Africa	~ 60 km	Chevallier and Woodford[2]
Paraná Basin, Brazil	~ 10 km	Schutter[3]
Neuquén Basin, Argentina	~ 20 km	Rossello et al.[4]
Rockall Trough, offshore Scotland	~ 3 km	Thomson and Hutton[5]
Faroe-Shetland Basin, North Sea	2 – 8 km	Smallwood and Maresh[6]
Offshore Senegal	300 m – 10 km	Rocchi et al.[7]
NW Australian shelf	~ 30 km	Symond et al.[8]
Nevada, USA	~ 1 km	Keating et al.[9]
Svalbard	~ 10 km	Senger et al.[10]
Mahad, India	~ 7 km	Duraiswami and Shaikh[11]
Tasmania	~ 6 km	Leaman[12]

References:

1. Polteau, S., Mazzini, A., Galland, O., Planke, S. and Malthe-Sørenssen, A. Saucer-shaped intrusions: Occurrences, emplacement and implications. *Earth and Planetary Science Letters*, **266,** 195–204 (2008).

2. Chevallier, L. and Woodford, A. Morpho-tectonics and mechanism of emplacement of the dolerite rings and sills of the western Karoo, South Africa. *South African Journal of Geology*, **102,** 43–54 (1999).

3. Schutter, S.R. Hydrocarbon occurrence and exploration in and around igneous rocks. *Geological Society, London, Special Publications*, **214,** 7–33 (2003).

4. Rossello, E.A., Cobbold, P.R., Diraison, M. and Arnaud, N. Auca Mahuida (Neuquén basin, Argentina): A Quaternary shield volcano on a hydrocarbon-producing substrate. *5th International Symposium on Andean Geodynamics, Extended Abstracts*, 549–552 (2002).

5. Thomson, K. and Hutton, D. Geometry and growth of sill complexes: insights using 3D seismic from the North Rockall Trough. *Bulletin of Volcanology*, **66,** 364–375 (2004).

6. Smallwood, J.R. and Maresh, J. The properties, morphology and distribution of igneous sills: modelling, borehole data and 3D seismic from the Faroe-Shetland area. *Geological Society, London, Special Publications*, **197,** 271–306 (2002).

7. Rocchi, S. et al. Detection of Miocene saucer-shaped sills (offshore Senegal) via integrated interpretation of seismic, magnetic and gravity data. *Terra Nova*, **19,** 232–239 (2007).

8. Symonds, P.A., Planke, S., Frey, O. and Skogseid, J. Volcanic evolution of the Western Australian continental margin and its implications for basin development. *The Sedimentary Basins of Western Australia*, **2,** 33–54 (1998).

9. Keating, G.N., Geissman, J.W. and Zyvoloski, G.A. Multiphase modeling of contact metamorphic systems and application to transitional geomagnetic fields. *Earth and Planetary Science Letters*, **198,** 429–448 (2002).

10. Senger, K., Planke, S., Polteau, S., Ogata, K. and Svensen, H. Sill emplacement and contact metamorphism in a siliciclastic reservoir on Svalbard, Arctic Norway. *Norwegian Journal of Geology/Norsk Geologisk Forening*, **94** (2014).

11. Duraiswami, R.A. and Shaikh, T.N. Geology of the saucer-shaped sill near Mahad, western Deccan Traps, India, and its significance to the flood basalt model. *Bulletin of volcanology*, **75,** 731 (2013).

12. Leaman, D.E. Form, mechanism, and control of dolerite intrusion near Hobart, Tasmania. *Journal of the Geological Society of Australia*, **22,** 175–186 (1975).

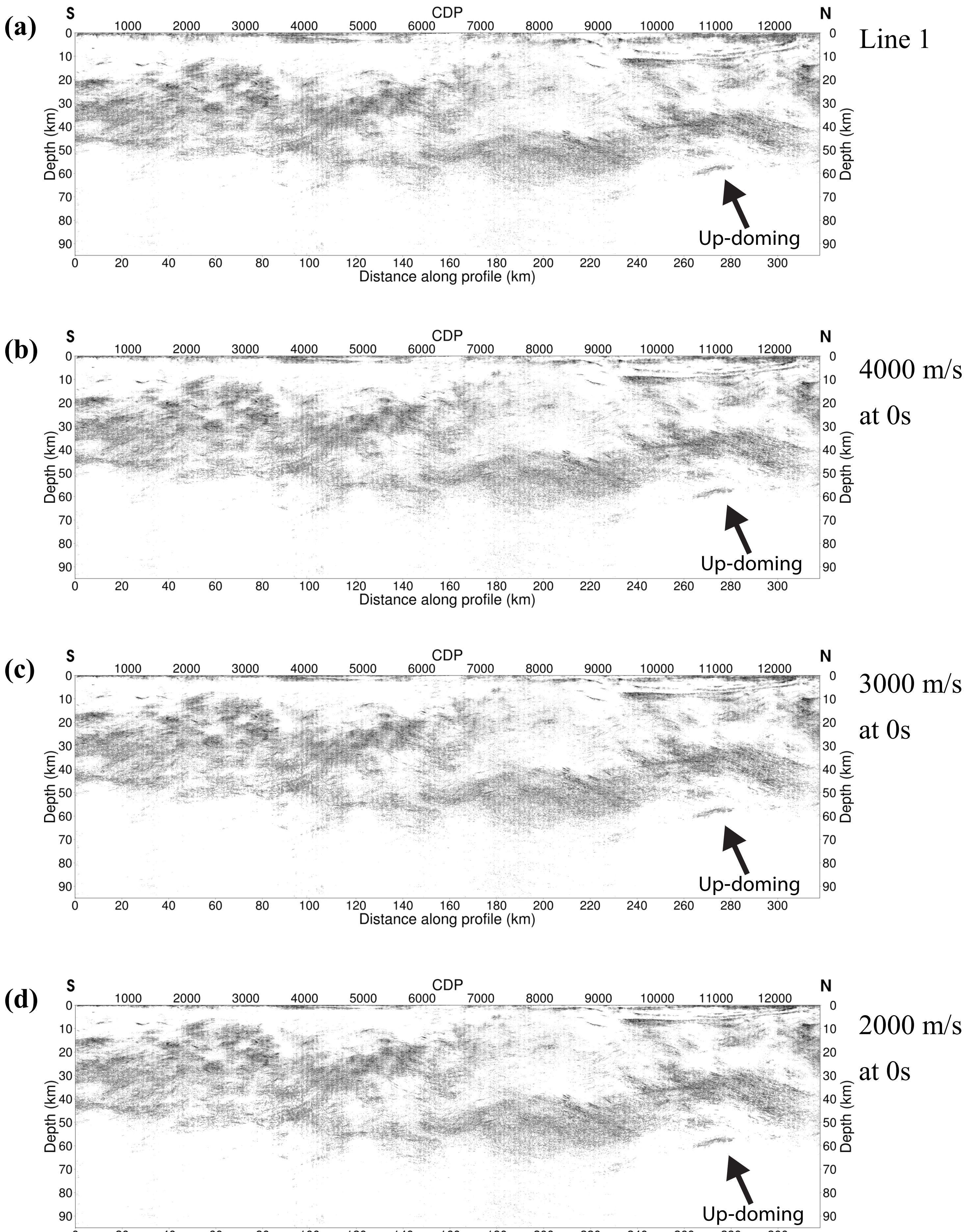
(a)
Line 1
(b)
4000 m/s
at 0s
(c)
3000 m/s
at 0s
(d)
2000 m/s
at 0s
S
N
CDP
1000
2000
3000
4000
5000
6000
7000
8000
9000
10000
11000
12000
Depth (km)
0
10
20
30
40
50
60
70
80
90
Distance along profile (km)
0
20
40
60
80
100
120
140
160
180
200
220
240
260
280
300
Up-doming

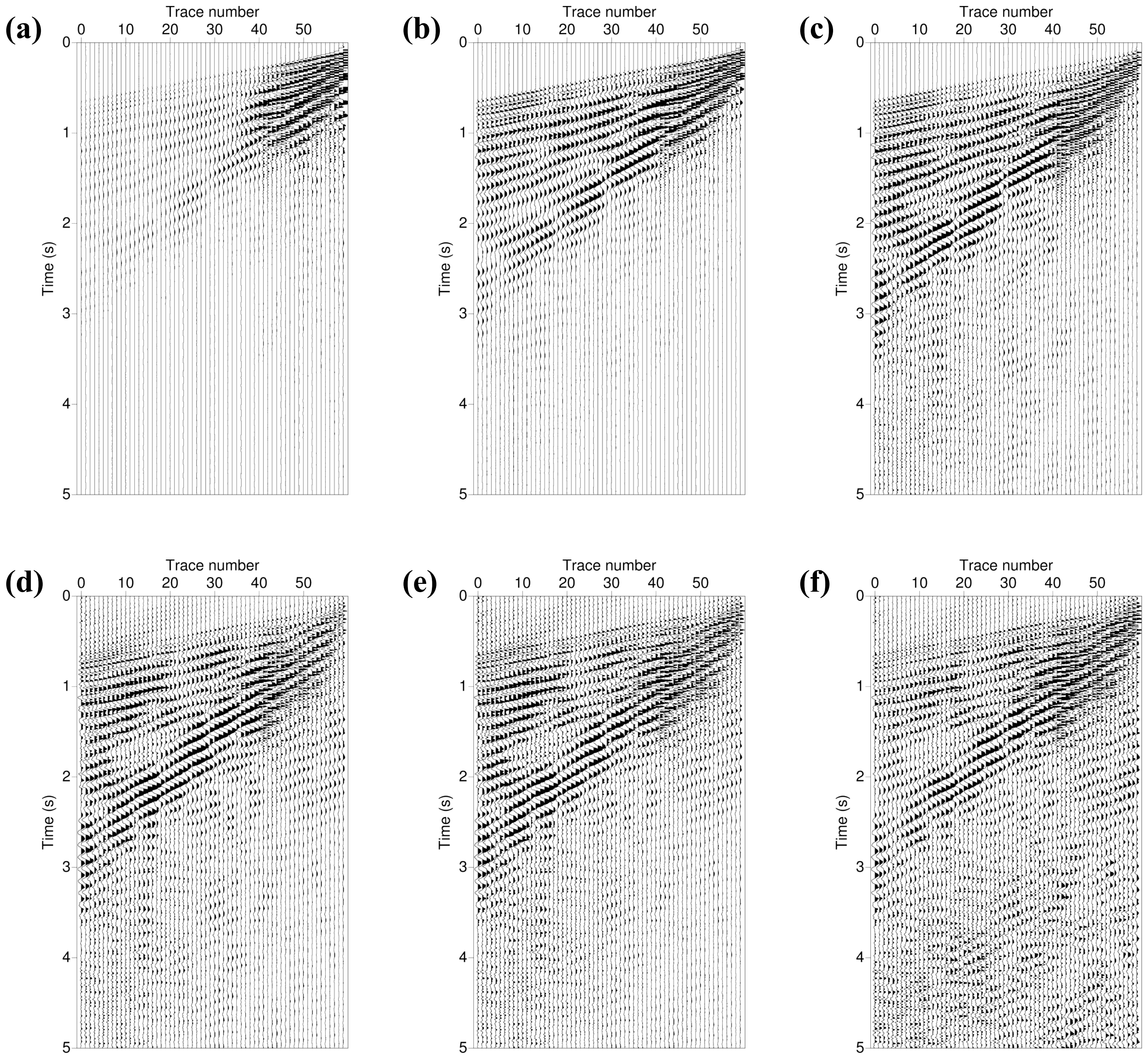

(a)
(b)
(c)
(d)
(e)
(f)
Trace number
Time (s)
0
10
20
30
40
50
0
1
2
3
4
5

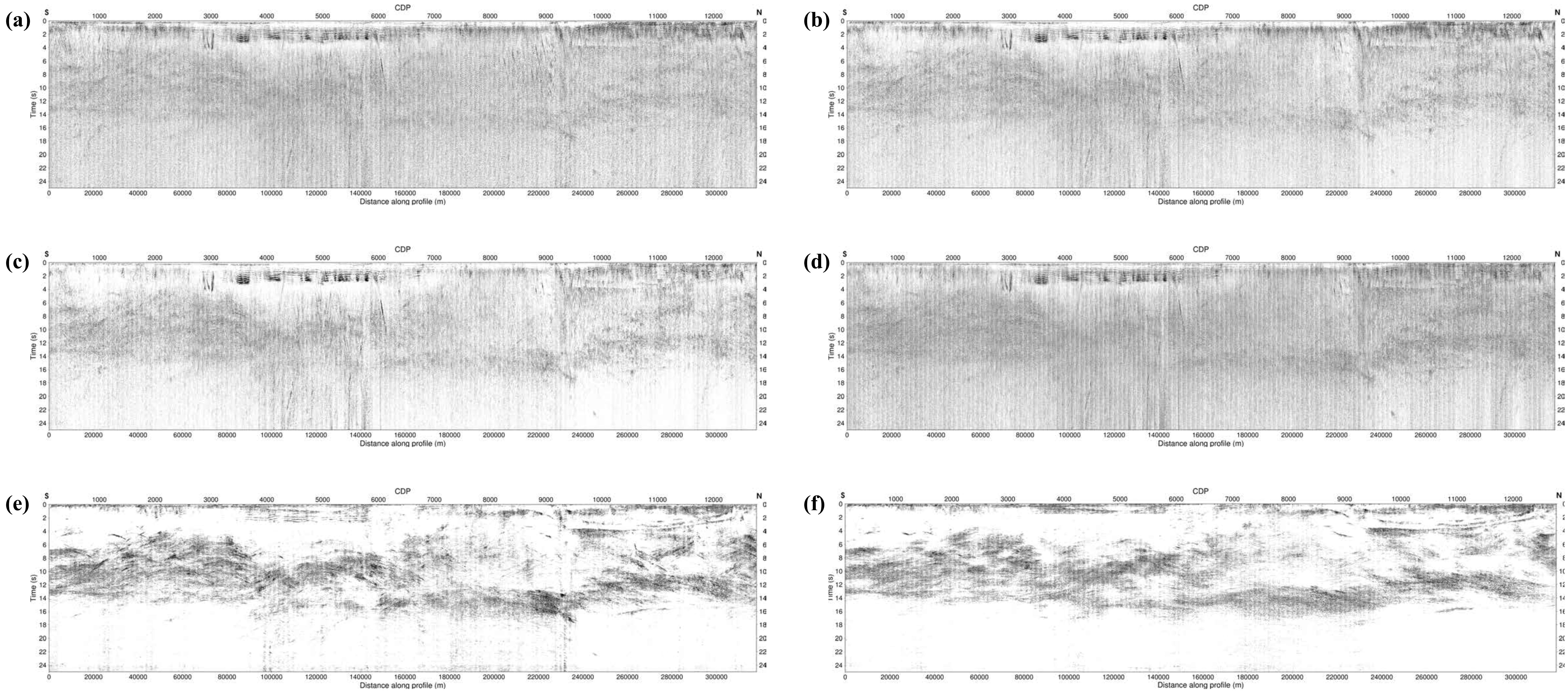
(a)
(b)
(c)
(d)
(e)
(f)
S
N
CDP
Time (s)
Distance along profile (m)

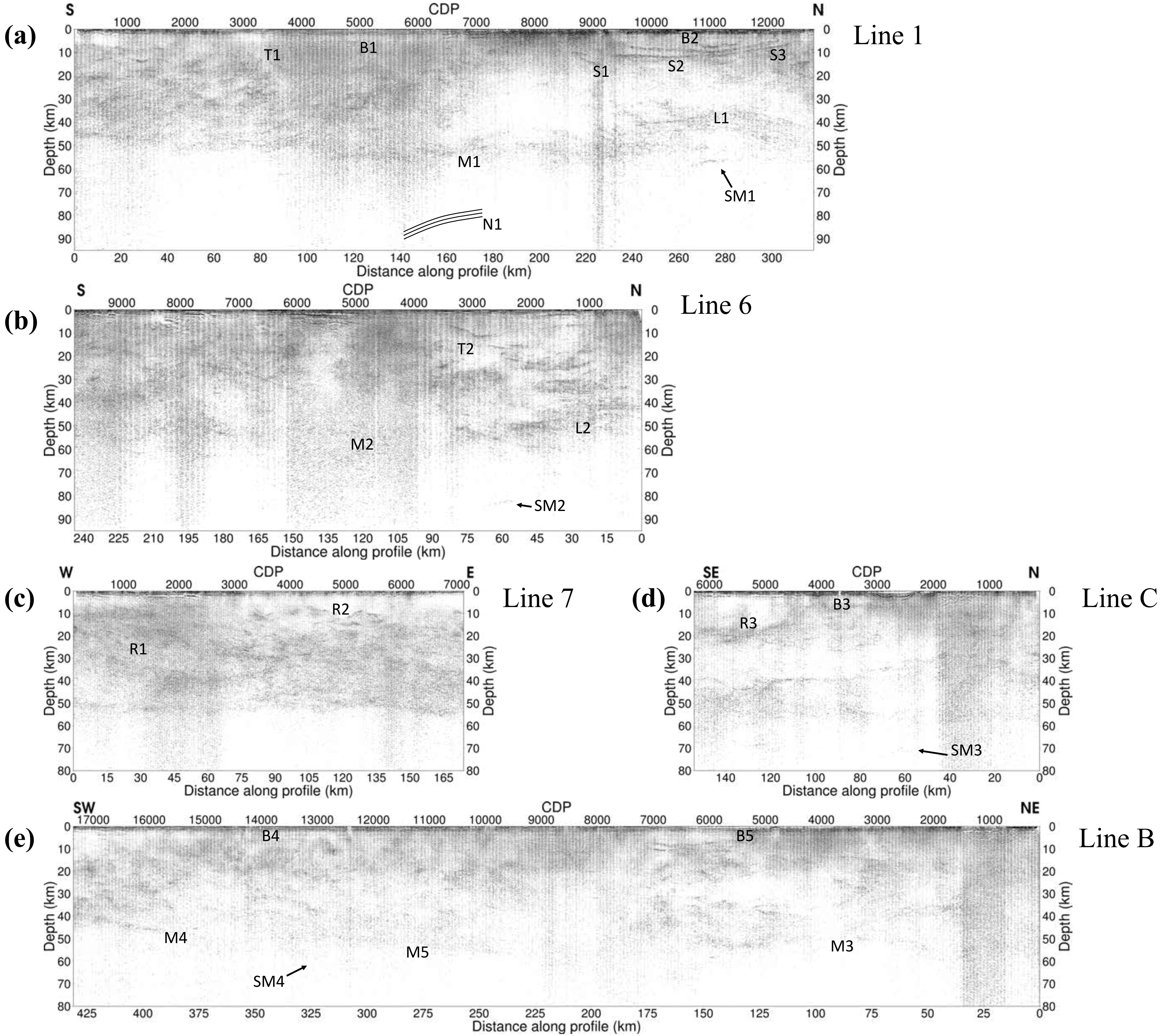
(a)
Line 1
S
N
CDP
Depth (km)
Distance along profile (km)
T1
B1
B2
S1
S2
S3
L1
M1
SM1
N1
(b)
Line 6
S
N
CDP
Depth (km)
Distance along profile (km)
T2
L2
M2
SM2
(c)
Line 7
W
E
CDP
Depth (km)
Distance along profile (km)
R1
R2
(d)
Line C
SE
N
CDP
Depth (km)
Distance along profile (km)
R3
B3
SM3
(e)
Line B
SW
NE
CDP
Depth (km)
Distance along profile (km)
B4
B5
M3
M4
M5
SM4

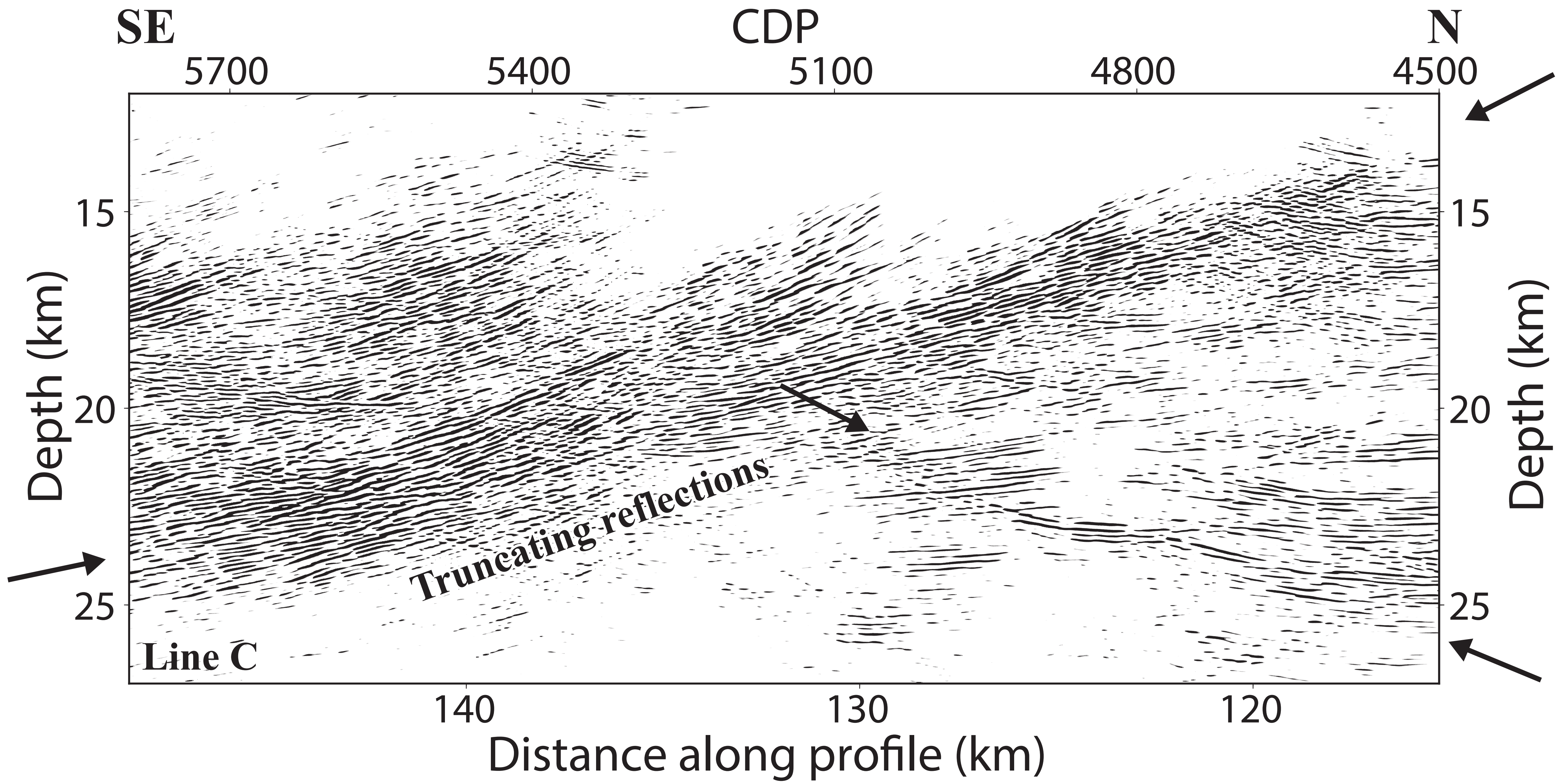

SE
CDP
N
5700
5400
5100
4800
4500
15
20
25
Depth (km)
Truncating reflections
Line C
140
130
120
Distance along profile (km)

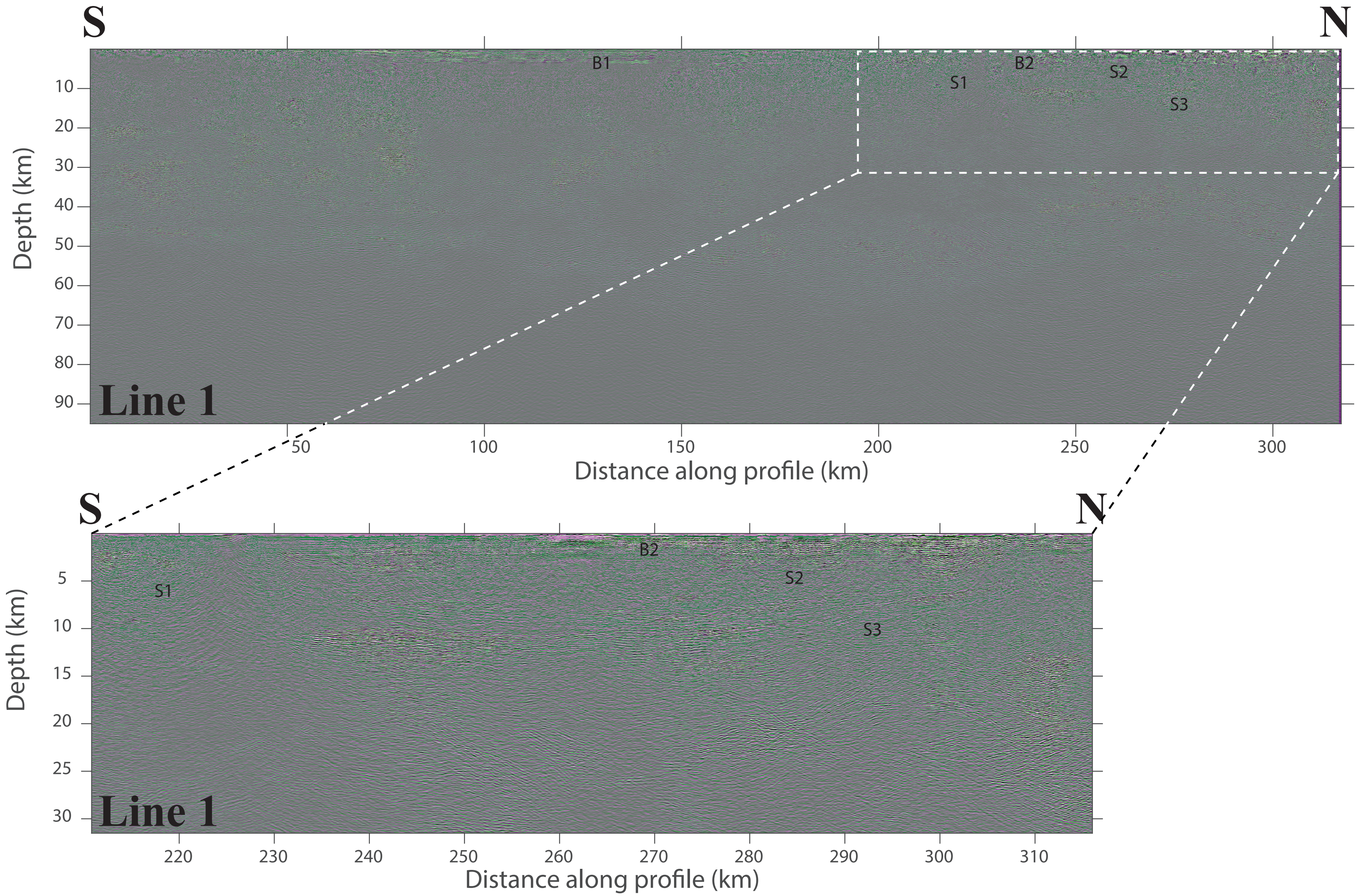

S
N
B1
B2
S1
S2
S3
Depth (km)
10
20
30
40
50
60
70
80
90
Line 1
50
100
150
200
250
300
Distance along profile (km)
S
N
B2
S1
S2
S3
Depth (km)
5
10
15
20
25
30
Line 1
220
230
240
250
260
270
280
290
300
310
Distance along profile (km)

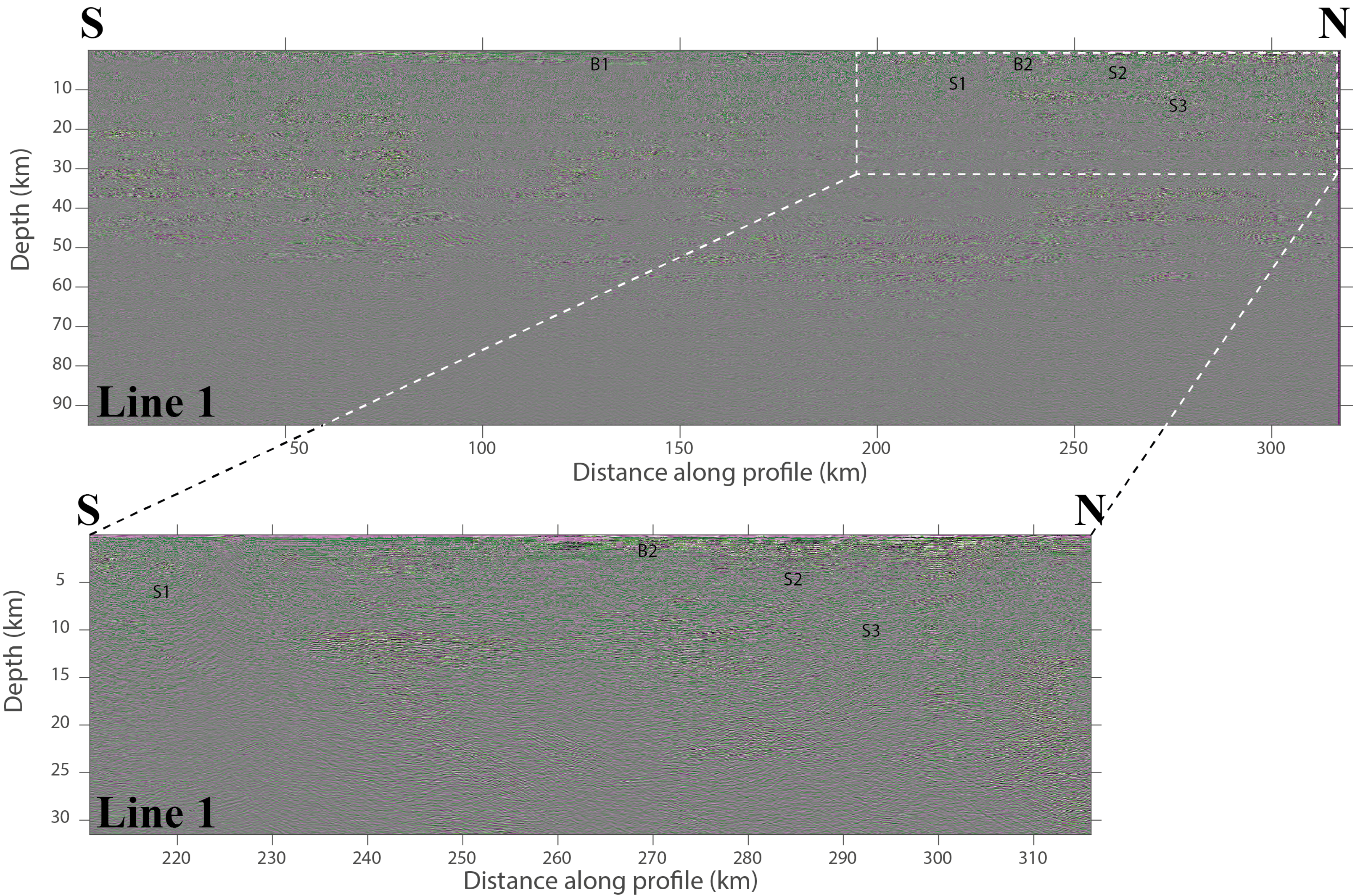
S
N
B1
B2
S1
S2
S3
Depth (km)
Distance along profile (km)
Line 1
S
N
B2
S1
S2
S3
Depth (km)
Distance along profile (km)
Line 1
